# Comparative study on the microbiota of colostrum and nipple skin from lactating mothers separated from their newborn at birth in China

**DOI:** 10.3389/fmicb.2022.932495

**Published:** 2022-10-03

**Authors:** Yanli Du, Qing Qiu, Jing Cheng, Zhili Huang, Ruixia Xie, Lu Wang, Xiangyu Wang, Zongli Han, Gang Jin

**Affiliations:** ^1^School of Medical Technology and Nursing, Shenzhen Polytechnic, Shenzhen, China; ^2^Department of Women Health Care, Shenzhen Luohu Maternity and Child Healthcare Hospital, Shenzhen, China; ^3^Department of Obstetrics, The University of Hong Kong-Shenzhen Hospital, Shenzhen, China; ^4^Delivery Center, Shenzhen Maternity and Child Healthcare Hospital, Shenzhen, China; ^5^Shenzhen Second People’s Hospital, Department of Gastroenterology, The First Affiliated Hospital of Shenzhen University, Shenzhen, China; ^6^Department of Neurosurgery, Peking University Shenzhen Hospital, Shenzhen, China

**Keywords:** microbiota, diversity, colostrum, nipple skin, mother–infant separation

## Abstract

Increasing studies have found breast milk (BM) contains its own microbiota. However, the route through which microbes enter the BM is still unclear. In order to verify the entero-mammary pathway of BM, we designed a rigorous study that prevented oral bacteria from contaminating the breast and nipple skin (NS) during baby nursing. Thirty-one healthy, postpartum mothers living in southern China who were immediately separated from their newborn after delivery were enrolled in this study. Using an aseptic protocol for sampling, sterile water was used to wash the NS and was then collected. Then the first drop of BM was discarded and colostrum was collected manually. Amplicon sequencing was performed targeting the V3–V4 region of the bacterial 16S rRNA gene, and the differences between the microbiota of the colostrum and NS were analyzed. Additionally, the effects of environmental factors, such as the delivery mode and intrapartum antibiotic exposure, on the diversity of the colostrum microbiota were also analyzed. We found significant differences in the α diversity and richness between the BM and NS as evidenced by richness, Chao1, and Simpson indices. There were 170 operational taxonomic units (OTUs) shared by colostrum and NS, while 111 and 87 OTUs were unique, respectively, as well as a clear distinction in OTUs was observed by unifrac binary analysis between them. Linear discriminant analysis effect size analysis found that anaerobes, such as *Bifidobacterium* and *Pantoea* at the genus level and enterobacteria including Enterobacteriaceae at the family level, were predominant in the colostrum, while the predominant bacteria on the NS were *Bacteroides, Staphylococcus*, and *Parabacteroides* at the genus level. BM is colonized by bacteria prior to baby suckling, and the diversity of the colostrum microbiota differs from that of the NS. The predominant microbiota taxa in BM indicated that they were likely to be transferred to the breast through the intestinal tract. Our study provides direct evidence for the revolutionary active migration hypothesis. Additionally, factors like intrapartum antibiotic exposure did not significantly affect the diversity of the microbiota in the BM. Therefore, it is suggested that mothers continue to provide BM for their newborns during separation.

## Introduction

Human breast milk (BM) is the best nutrition for infants, as it provides essential nutrients, immune cells, and bioactive components. Breastfeeding is associated with considerable health benefits for infants, including protection from diarrhea (Duijts et al., 2010), necrotizing enterocolitis (Herrmann and Carroll, 2014), respiratory infections (Lanari et al., 2013; Tromp et al., 2017), as well as obesity, type 2 diabetes, and cardiovascular disease (Arenz et al., 2004). These benefits are, especially, relevant to infants with increased susceptibility to infections, such as preterm or sick infants. Additionally, increasing evidence has shown that BM contains its own microbiota, which is important for maintaining both mammary and infant health (Zimmermann and Curtis, 2020). Breastfeeding was considered to be a smart investment in people and in economies (Hansen, 2016), thus World Health Organization (WHO) currently recommends an exclusively breastfed diet until 6 months of age, with solid food and breast milk continuing thereafter (WHO, 2001).

In recent years, the study of the BM microbiota has become increasingly extensive. BM is recognized as a source of commensal and potentially probiotic bacteria (Jost et al., 2014) and may also influence both neonatal gut colonization (Gueimonde et al., 2007) and the maturation of the immune system (Jost et al., 2014). In the past, bacteria isolated from BM were thought to be a contaminant from the infant’s oral cavity, mother’s skin, or due to incorrect handling or processing (Heikkila and Saris, 2003). One study estimated that average consumption of 800 ml of BM per day correlates to an infant ingesting approximately 8 × 10^7^ to 10^10^ bacteria per day (Zimmermann and Curtis, 2020). Traditional culture and isolation techniques and culture-independent molecular methods, such as quantitative PCR, cloning and sequencing of bacterial 16S rRNA gene fragments (Cabrera-Rubio et al., 2012, 2016; Jost et al., 2013; Sakwinska et al., 2016; Ruiz et al., 2019), and metagenomics shotgun sequencing (Ward et al., 2013; Jimenez et al., 2015), have revealed the diversity and complexity of bacteria in BM.

The origin of the BM microbiota is the subject of much debate. There are currently two hypotheses regarding the source of the BM microbiota: the traditional pollution theory and the active migration theory. According to pollution theory, the microorganisms in breast milk come from the mother’s nipple and areola and the baby’s mouth. During the process of maternal feeding, the negative pressure of sucking generated by the infant allows the microorganisms colonizing the skin surrounding the nipple and areola to enter the mammary gland along the breast tube, or the newborn’s mouth is contaminated by microorganisms from the mother’s intestinal tract and vagina during delivery, which enter the mammary gland during feeding. Other studies have suggested that the microbiota in the mother’s intestines and vagina can naturally “migrate” to the newborn’s intestines during delivery (Martin et al., 2007a; Makino et al., 2011; Sanz, 2011).

The other is the hypothesis that maternal bacteria translocate through the intestinal epithelial barrier, migrate to the mammary glands *via* an endogenous cellular route, such as a bacterial entero-mammary pathway, and subsequently colonize the gut of the breastfed neonate (Martin et al., 2007b; Jost et al., 2014).

In order to test the hypothesis regarding an entero-mammary pathway of microbiota translocation in breast milk, we washed the skin surrounding the nipple and collected the cleaning fluid, in addition to the colostrum, from 31 mothers who were separated from their infants immediately after delivery. We used 16S rRNA gene sequencing to detect and analyze differences between the microbiota profile of the BM and nipple skin. We also investigated the impact of newborn gender, delivery mode, parity, gestational age, and intrapartum antibiotic exposure on the microbiota composition of colostrum.

## Materials and methods

### Participants

The study was conducted from January to February 2021 in the Luohu Maternal and Child Hospital in Shenzhen, China. Thirty-one healthy volunteers who met the inclusion and exclusion criteria were included in the study. The inclusion criteria were as follows: 20–45 years old, healthy during pregnancy, without any common pregnancy complications, did not use probiotics during pregnancy, settled in Shenzhen, the Han nationality, and normal lactating woman. Exclusion criteria included pregnant women with gestational diabetes, pregnancy-induced hypertension syndrome, acute communicable diseases, and ethnic minorities. After birth, the newborns were immediately transferred to the neonatal intensive care unit (NICU) for monitoring or treatment due to premature rupture of membranes or other reasons, resulting in the separation of mother and baby. Thus, the newborn did not nurse from the mother’s breast immediately after delivery. Further, BM samples were collected only from mothers who are yet to nurse their newborn babies. The postpartum mothers were usually hospitalized for 3−5 days and then discharged with their baby or discharged before the baby. In order to ensure the samples were all in the same ward environment, we collected BM from lactating mothers during their hospitalization. Since we collected BM within 5 days after delivery, the milk collected was colostrum. Additionally, mothers generally did not pump BM before delivery.

The participants completed a questionnaire that included basic maternal information (name, age, telephone number, and address), pregnancy and childbirth information (number of pregnancies, parity, the number of gestational weeks at delivery, education level, pre-pregnancy weight, pre-partum weight, breast changes during pregnancy, diet, delivery method, and prophylactic use of antibiotics before or after delivery), and information about the infant, including gender, height, and weight, as well as the reason for separation from the mother at birth (Table 1 and Supplementary Table S1).

**TABLE 1:** Average demographics of the participants.

Variable	Data
Mother age (years), Mean (SD)	29.3 (4.6)
Mother gain weight during pregnancy (kg), Mean (SD)	13.0 (4.5)
Pregnancy number, X (*N*)	1 (21)
	2 (7)
	3 (3)
Gestational age (weeks), w (*N*)	37–42 w (24)
	< 37 w (7)
Delivery mode (*N*)	Vaginal (17)
	C-section (14)
Intrapartum antibiotics during delivery (*N*)	Yes (22)
	No (9)
Infant gender (*N*)	Male (19)
	Female (12)
Newborn weight (kg), Mean (SD)	3.0 (0.6)
Sampling days after birth (days), Mean (SD)	3.0 (1.1)

### Sample collection

During hospitalization, colostrum was collected from all 31 lactating mothers within 5 days after delivery. The first BM and NS samples of the mother were collected on 5 January 2021, and the last samples were collected on 25 January 2021. Colostrum was collected by an International Board Certified Lactation Consultant (IBCLC) in our study group, and her assistant using an aseptic protocol. Briefly, 8–10 ml of sterile water was used to wash one nipple and the surrounding nipple skin (NS). The sterilized water for rinsing the nipple skin was collected in an enzyme-free, aseptic centrifugal tube. The first drop of breast milk was discarded with an aseptic yarn block, and the colostrum (3–5 ml) was manually collected into an enzyme-free, aseptic centrifugal tube by researchers with sterile gloves. After sealing the tube with sealing film, the BM and NS samples were quickly frozen in liquid nitrogen and then transferred to a −80°C freezer for storage. The total genomic DNA was extracted on 19th February 2021. These processes prevented the microbiota from replicating.

### DNA extraction and PCR amplification

The bacterial DNA was extracted from the BM and lotion samples using the TGuide S96 Magnetic Soil/Stool DNA Kit [Tiangen Biotech (Beijing) Co., Ltd., China], and PCR amplification was conducted with barcoded-specific bacterial primers targeting the variable region 3–4 (V3–V4) of the 16S rRNA gene. The primers used were 335F: 5′-CADACTCCTACGGGAGGC-3′ and 769R: 5′-ATCCTGTTTGMTMCCCVCRC-3′.

The PCR was performed in a total reaction volume of 10 μl: DNA template (5−50 ng), *Vn F (10 μM, 0.3 μl), *Vn R (10 μM, 0.3 μl), KOD FX Neo Buffer (5 μl), dNTP (2 mM, 2 μl), KOD FX Neo (0.2 μl), and ddH2O (up to 10 μl). The amplification conditions were as follows: an initial denaturation at 95°C for 5 min, followed by 25 cycles of 95°C for 30 s, 50°C for 20 s, and 72°C for 40 s, and a final extension at 72°C for 7 min. The PCR amplified products were mixed and purified by an Omega DNA purification column (Norcross, GA, United States). The mixed PCR amplified products were then purified and recovered using 1.8% agarose gel electrophoresis.

### Processing of the sequencing data

Construction of sequencing libraries and paired-end sequencing was performed on an Illumina NovaSeq6000 platform at Biomarker Technologies Co., Ltd. (Beijing, China) according to standard protocols. Paired-end reads were merged using FLASH v1.2.7 (Yang et al., 2020), and tags with more than six mismatches were discarded. The merged tags with an average quality score < 20 in a 50-bp sliding window were determined using Trimmomatic (Sheng et al., 2019), and those shorter than 350 bps were removed. Possible chimeras were further removed, and the denoised sequences were clustered into operational taxonomic units (OTUs) with 97% similarity using USEARCH (version 10.0) (Edgar, 2010), and the OTUs with reabundance < 0.005% were filtered. Taxonomy was assigned to all OTUs by searching against the Silva databases (Release128) using USEARCH software (version 10.0). We used Decontam (Version 0.0.1) to minimize the sources of probable external contamination.

### Statistical analysis

The statistical analyses were performed in R (Version 4.0.1). Microbiota profiles were included in the estimation of alpha diversity (referring to diversity within a particular region or ecosystem) and beta diversity (comparing the similarity of species diversity among different samples) analyses as described by Liu et al. (2021). We obtained the richness, chao1, and Shannon indices of alpha diversity and compared these differences between BM and NS by LSD *t*-test.

Dissimilarities between BM and NS were estimated with the Bray–Curtis dissimilarity index and Unifrac indices (Lozupone et al., 2007) and analyzed with principal coordinate analysis (PCoA). Moreover, permutational multivariate analysis of variance was used to describe the strength and significance that a categorical factor has in determining the variation of ecological distances. The differential OTU abundance and taxa were analyzed by Wilcoxon rank-sum tests in R version 4.0.1 (FDR < 0.05, the mean relative abundance > 0.1%).

Furthermore, we employed linear discriminant analysis (LDA) effect size (LEfSe; Segata et al., 2011) to identify the significant taxonomic difference among influencing groups, with edgeR (Robinson et al., 2010) for verification if necessary. A logarithmic LDA score of 2.0 was set as the threshold for discriminative features. Adobe Illustrator was used to plot the results.

### Ethics statement

This study was conducted according to the guidelines set forth in the Declaration of Helsinki. The experiments in this study were approved by the Ethics Committee of the Luohu Maternal and Child Health Hospital (No. LL2022051337). Written consent was obtained from each volunteer.

## Results

### Participants’ characteristics

The study was conducted on samples collected from 31 healthy mothers (aged 21–42 years) who were separated from their infants immediately after delivery without skin-to-skin contact with babies. The causes of mother-infant immediate separation after delivery included neonatal factors, such as pulmonary sequestration, premature birth, and amniotic fluid inhalation, and maternal factors, such as premature rupture of membranes, placental abruption, and extended vaginal delivery. Details are presented in Table 1 and Supplementary Table S1. The average age of mothers was 29.3 ± 4.6 years. Nearly, 68% of mothers gave birth to their first child, 23% for the second baby, and less than 9% for the third. Among the 31 newborns, 19 were male and 12 were female, and 17 were delivered vaginally and 14 were born by cesarean section (C-section). The average birth weight of babies was 3.0 ± 0.6 Kg. Twenty-two mothers received intrapartum antibiotic prophylaxis (IAP) through intravenous infusion, including 14 who delivered *via* C-section and 7 who delivered vaginally. The main antibiotics were cefazolin sodium, which is a first-generation cephalosporin, and cefuroxime sodium, which is a second-generation cephalosporin. All 31 normal lactating mothers lived in Shenzhen, south of China, and experienced anxiety after being separated from their newborns.

### Microbiota profiling by sequencing of 16S rRNA gene amplicons

The quantity of microbiota in the BM and NS samples collected *via* an aseptic protocol was sufficient for microbiota profiling based on 16S rRNA gene sequencing. All 62 samples, which included 31 BM and 31 NS samples, yielded quantifiable PCR products and were sequenced. The analysis resulted in the generation of a total of 4,646,066 clean reads, representing an average of 74,937 clean reads per sample. The average reads were 78,158 per sample in the BM samples and 71,714 reads per sample in the NS samples. Reads were clustered at the similarity level of 97.0%, and OTUs were obtained. Totally, 11,190 OTUs were retained for diversity analyses, with an average OUT abundance of 243 in BM and 212 in NS.

### Alpha diversity analysis of the microbiota in the breast milk and nipple skin

**FIGURE 1:**
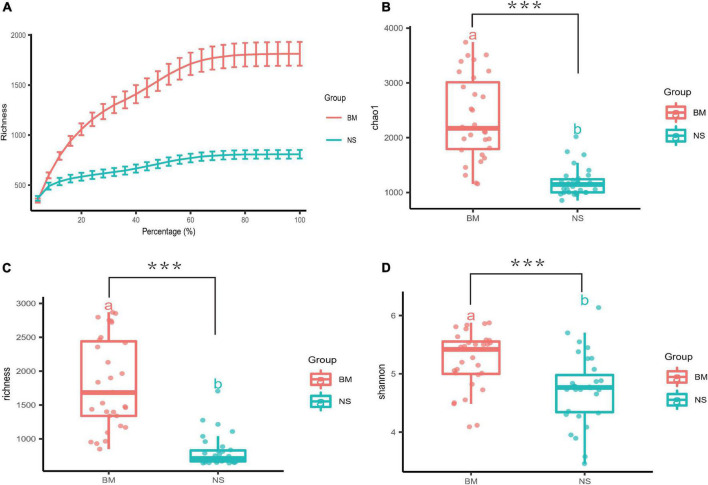
Measures of α diversity for BM and NS. **(A)** A rarefaction curve was used to evaluate the sequencing saturation of the samples, and it showed the mean value and standard error of the sequencing depth and the quantity of OTUs. The smoothing of the curves indicated that the sequencing was saturated. **(B–D)** Box plots of three different α diversity measures: Chao1, richness, and Shannon. The three indices were based on OTUs clustered at 97% similarity for the BM and NS samples (BM, *n* = 31 vs. NS, *n* = 31). There were significant differences in Chao1, richness, and Shannon between BM and NS groups (a and b above boxes represent statistical differences) with FDR-adjusted *p* = 0.000, 0.001, and 0.001 with LSD *t*-test, respectively. Each scatter represents a sample. The horizontal bars within boxes represent the median, and the tops and bottoms of the boxes represent the 75th and 25th quartiles, respectively. ***p ≤ 0.001.

A dilution curve (Wang et al., 2012) (rarefaction curve) randomly takes a certain number of sequences from a sample, counts the number of species represented by the sequences, and constructs a curve based on the number of sequences and species. This curve is then used to verify whether the amount of sequencing data is sufficient to reflect the species diversity in the sample, and indirectly reflects the species richness in the sample. The rarefaction curves of the OTUs from the BM and NS samples approached the plateau phase with more than 40,000 sequences per sample, indicating that these values were saturated and there was no need to determine more sequences (Figure 1A). The α diversity of the microbiota from the BM and NS was estimated through sample richness (Figure 1C), Chao1 (Figure 1B), and Shannon index (Figure 1D). The BM samples displayed higher α diversity values than the NS samples, as determined *via* the three indices mentioned above (LSD *t*-test *p* = 0.000, 0.001, and 0.001, respectively) (Figures 1B–D). In short, the microbiota in BM and NS detected by 16S rRNA gene sequencing was diverse. Moreover, the microbial abundance in BM was higher than that in the NS.

### Analysis of taxa shared or differentially represented in breast milk and nipple skin samples

**FIGURE 2:**
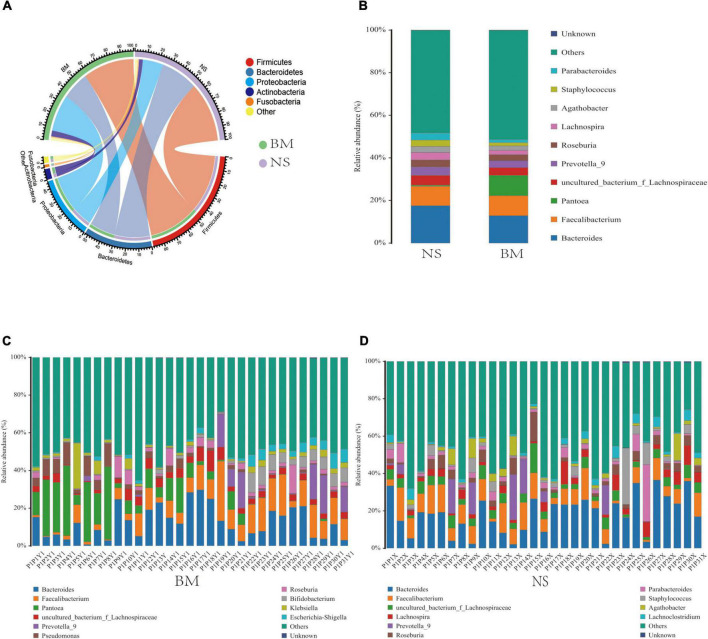
The common **(A,B)** and unique **(C,D)** relative abundances of predominant microbiota taxa in BM and NS. The common microbiota at the panels **(A)** phylum level and **(B)** genus level. The unique microbiota at the genus level of panels **(C)** BM and **(D)** NS. The proportion of microbial abundance was more than 0.1%, and the top 10 were displayed.

Focusing on the differences and similarities of specific phylum between the BM and NS microbiotas (Figure 2A), we found that Firmicutes was the dominant phylum in both sample types, followed by Bacteroidetes, Proteobacteria, Actinobacteria, and Fusobacteria. These phyla were detected across all the samples analyzed. Analysis at the genus level (the microbial relative abundance was above 0.1% and the top 10 were displayed) (Figure 2B) found that the common genera in both the BM and NS microbiotas were *Bacteroides, Faecalibacterium, Pantoea, uncultured_bacterium_f_Lachnospiraceae, Prevotella_9, Roseburia, Lachnospira, Agathobacter, Staphylococcus, Parabacteroides*, and others. On the other hand, there were also differences in the abundance of some genera between the BM and NS samples. *Faecalibacterium* (9.39% vs. 9.11%) and *Pantoea* (9.4% vs. 0.51%) were dramatically higher in the BM microbiota when compared to the NS microbiota, while *Lachnospira* (2.04% vs. 3.48%), *Staphylococcus* (1.52% vs. 3.03%), and *Parabacteroides* (1.30% vs. 3.12%) were dramatically lower in the BM microbiota when compared to the NS microbiota.

According to the microbial abundance top10 statistics, the unique bacterial genera of the BM samples included *Pantoea, Pseudomonas, Bifidobacterium, Klebsiella*, and *Escherichia-Shigella* (Figure 2C), which were higher than 0.1%. The unique bacterial genera in the NS samples were *Lachnospira, Parabacteroides, Staphylococcus, Agathobacter*, and *Lachnoclostridium* (Figure 2D). Additionally, the shared genera between the BM and NS microbiotas were *Bacteroides, Faecalibacterium, uncultured_bacterium_f_Lachnospiraceae, Prevotella_9, Roseburia*, and others (Figures 2C,D). Additionally, high variability in the microbiota composition of both BM and NS was observed among the 31 mothers. Thus, the bacterial communities were generally complex and showed individual-specific profiles.

### Beta diversity analysis of the microbiota in the breast milk and nipple skin

**FIGURE 3:**
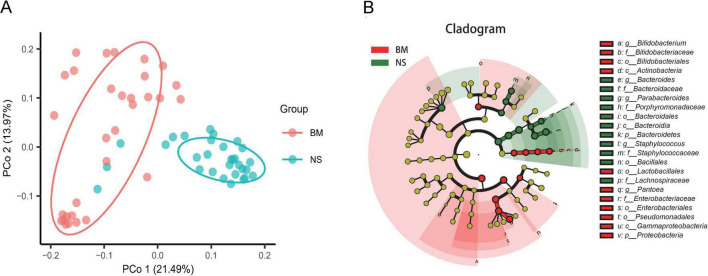
Differences in the microbiota between BM (colostrum) and NS. **(A)** Unconstrained principal coordinate analysis with Bray–Curtis distance showing that the BM microbiota separated from those of NS in the first axis (21.49% of variance explained, *P* < 0.001, permutational multivariate analysis of variance (PERMANOVA) by Adonis; *n* = 31 in each group). Ellipses cover 68% of the data for BM and NS samples. The percentage of variation indicated in each axis corresponds to the fraction of the total variance explained by the projection. **(B)** Taxonomic cladogram from linear discriminant analysis (LDA) effect size (LefSe) showing microbiota differences in BM and NS taxa. Dot size is proportional to the abundance of the taxon. The yellow circles represent the classification with no significant difference. P, phylum; c, class; f, family; o, order; g, genus.

We examined differences in the microbiota of BM and NS at the OTU level. First, a Venn diagram that showed 170 overlapped OTUs between BM and NS was constructed. The unique OTUs were 111 for BM and 87 for NS (Supplementary Figure S1A). Through differential abundance analysis, 77 OTUs were depleted and 107 were enriched in the BM when compared with the NS (Supplementary Figure S1B). We found that the composition of the bacterial microbiota of BM was different from that of NS. Unifrac binary analysis (PCoA) of Bray–Curtis distance revealed that the microbiota of BM and NS formed two distinct clusters, which separated along the first coordinate axis (Figure 3A), indicating that the BM microbiota differed from the NS microbiota.

We then analyzed the enrichment of OTUs according to their taxonomy using Manhattan plots (Supplementary Figure S1C). Compared with the NS, OTUs enriched in the BM belonged to the genera *Prevotella and Erwinia*, while OTUs depleted in the BM were commonly in the genera *Bacteroides, Phocaeicola, Staphylococcus, Agathobacter*, and *Kineothrix* (FDR < 0.05, Wilcoxon rank-sum test; Supplementary Figure S1C). Additionally, the LEfSe analysis including taxonomic cladogram (Figure 3B) and linear discriminant analysis (LDA) scores (LDA values higher than 4.0, *p* = 0.05) (Supplementary Figure S1D) also determined microbiota unique to the BM, including *Bifidobacterium* at the genus level, *Enterobacteriaceae* at the family level, *Lactobacillales* and *Pseudomonadales* at the order level, *Actinobacteria* and *Gammaproteobacteria* at the class level, and *Proteobacteria* at the phylum level, which was totally different from the NS. The unique NS microbiota included *Bacteroides, Staphylococcus*, and *Parabacteroides* at the genus level, *Lachnospiraceae, Bacteroidaceae*, and *Porphyromonadaceae* at the family level, and *Bacillales* at the order level.

### Analysis of various factors may influence the colostrum (breast milk) microbiota diversity

**FIGURE 4:**
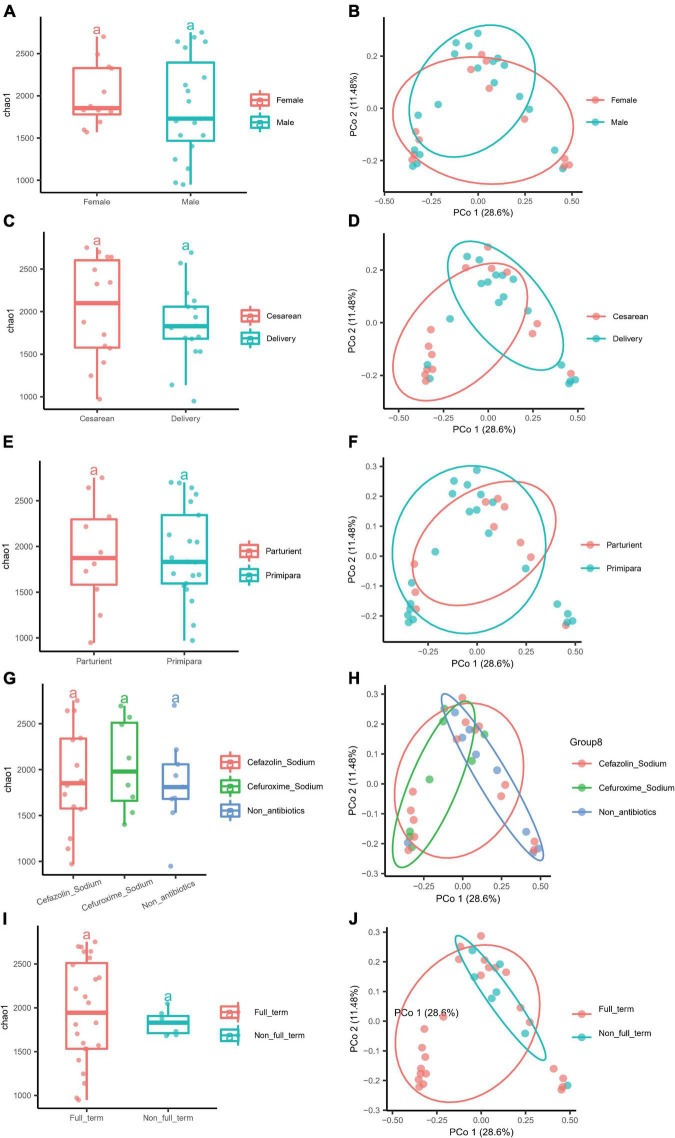
Different factors had no influence on the α and β diversity of the microbiota of BM. **(A)** The α diversity of newborn gender (Female and Male). **(B)** The β diversity of newborn gender (Female and Male). **(C)** The α diversity of the delivery mode (Cesarean and Delivery). **(D)** The β diversity of the delivery mode (Cesarean and Delivery). **(E)** The α diversity of parity (Parturient and Primipara), and **(F)** the β diversity of parity (Parturient and Primipara). **(G)** The α diversity of intrapartum antibiotic prophylaxis (Cefazolin sodium, Cefuroxime sodium, and Non-antibiotics), and **(H)** the β diversity of intrapartum antibiotics prophylaxis (Cefazolin sodium, Cefuroxime sodium, and Non-antibiotics). **(I)** The α diversity of gestational age (Full-term and Non-full-term). **(J)** The β diversity of gestational age (Full-term and Non-full-term).

Possible factors, such as the parity, delivery method, IAP, gestational age, and gender of the newborn, that might affect the microbiota diversity were further explored. On the other hand, we tried to apply LEfSe to analyze the characteristic microbiota taxa for different influencing factors and verify them using edgeR for subsequent studies on the characteristic microbiota. Only taxa with LDA values higher than 3.0 were presented (Supplementary Figure S2), and the LDA value for each lineage was listed. The gender of the newborn had no significant effect on the α and β diversity values of the BM microbiota (Figures 4A,B). The LEfSe analysis showed that the BM of female newborns was rich in *Streptococcaceae* at the family level, while the BM of male newborns was rich in *Roseburia* and *Alcaligenaceae* (Figure 4A). In addition, the edgeR verified that *Roseburia* was enriched at the genus level (data not shown). Parity had no significant effect on the α and β diversity values of the BM microbiota (Figures 4E,F). Also, the delivery mode had no significant effect on the α and β diversity of the BM microbiota (Figures 4C,D). However, LEfSe analysis showed that the BM microbiota from mothers who delivered *via* CS was rich in *Bifidobacterium* (Figure 4B), which was supported by edgeR (data not shown). IAP during delivery also had no significant effect on the α and β diversity of the BM microbiota. Moreover, the two antibiotics administered to mothers (cefazolin sodium and cefuroxime sodium) had no significant effect on the microbiota diversity of BM (Figures 4G,H). However, LEfSe analysis found that mothers who did receive IAP had BM that was rich in *Lachnospiraceae* at the genus level, which was verified by edgeR. Those who did not receive IAP were rich in *Lactobacillus* in BM (Supplementary Figure S2C). Gestational age also had no significant effect on the α and β diversity of the BM microbiota (Figures 4I,J). However, mothers who delivered a full-term infant(s) had BM that was rich in *Bifidobacteria*, as verified by LEfSe and edgeR (Supplementary Figure S2D).

## Discussion

The ideal and most common state for mother and newborn is to be together, that is, the infant is immediately placed on the mother’s skin after birth and encouraged to nurse. This allows the newborn to consume BM, which provides a variety of nutrients that promote healthy programming (Cortes-Macias et al., 2021), as well as a multitude of immunomodulating components, such as soluble immunoglobulin A and growth factors that can reduce the risk of infectious diseases (Chi et al., 2021). However, not all mothers and newborns can be together after birth due to various factors, such as premature birth and amniotic fluid inhalation. Once the mother and baby are separated due to neonatal diseases or some other reasons in China, some mothers struggle to maintain and/or produce BM, resulting in a low supply of BM, which may affect the health of the newborn. The participants in this study were mothers who were separated from their newborns immediately after delivery. Their BM was sampled for studying the microbiota and exploring the origin of microbiota. The aim of this study was to provide a basis for continuous lactation in mothers separated from their newborns and encourage the mothers to provide BM to their newborns during separation.

### The breast milk (colostrum) and nipple skin microbiota in lactating mothers separated from their newborns at birth is diverse

Through breastfeeding, newborns and infants are exposed to microorganisms in BM, which stimulate the intestinal immune function of newborns and infants. The BM microbiota aids the development and functional maturity of the intestinal immune system and reduces the susceptibility to disease (Gollwitzer and Marsland, 2015). Thus, BM is recognized as a major force that shapes the infant gut microbiome in early life. Currently, little is known about the composition of the BM microbiota, and even less is known about the factors that determine it (Zimmermann and Curtis, 2020). To our knowledge, this is the first study to explore the source and composition of the colostrum (first stage of BM) microbiota in a large cohort of Southern Chinese mothers immediately separated from their newborns after delivery. We carefully designed and adopted an aseptic sampling protocol and sample preservation, as well as the elimination of the first drip, which included a few microliters to milliliters of BM, to avoid external contamination and ensure the accuracy and reliability of the results.

Colostrum, generally produced within the fifth day after delivery, is the first stage of BM during lactation. It is rich in proteins and minerals, and contains many immune-active substances, such as antibodies and complement factors (Ballard and Morrow, 2013). It has been reported that colostrum is also rich in microorganisms. Our study analyzed the microbiota of colostrum and NS from mothers who were immediately separated from their newborns after delivery. The rarefaction curves demonstrated that all BM and NS samples reached standard quality requirements for sequencing, and all samples were sequenced sufficiently (Figure 1A). Moreover, α diversity of the BM microbiota was assessed *via* richness, Chao1, and Shannon indices (Figures 1B–D). The dominant phyla in colostrum (BM) were *Firmicutes* and *Proteobacteria*, which was consistent with previous studies (Urbaniak et al., 2016; Biagi et al., 2017; Murphy et al., 2017; Pannaraj et al., 2017; Simpson et al., 2018; Hermansson et al., 2019; Moossavi et al., 2019). However, the next most abundant phyla were *Bacteroidetes, Actinobacteria*, and *Fusobacteria* (Figure 2A), which differed from previous reports.

At the genus level, the bacteria unique to the colostrum (BM) were *Pantoea, Pseudomonas, Bifidobacterium, Klebsiella*, and *Escherichia-Shigella* (Figure 2C vs. Figure 2D). The microbiota shared with the NS was *Bacteroide, Faecalibacterium, uncultured_bacterium_f_Lachnospiraceae, Prevotella_9*, and *Roseburia* (Figure 2B). These results differed from full-term newborns who routinely suck their mother’s breast after birth, which had higher relative abundances of *Weisella, Leuconostoc, Streptococcus* (Urbaniak et al., 2016), and *Lactococcus* (Cabrera-Rubio et al., 2012). Notably, the genus *Staphylococcus* has been reported to have higher relative abundances during the first 10 days of life (Cabrera-Rubio et al., 2012; Sakwinska et al., 2016; Simpson et al., 2018), in addition to being universally observed in mature BM (Solis et al., 2010; Jost et al., 2013) by pyrosequencing. However, in our study, *Staphylococcus* was found to be predominant in the NS microbiota (3.03%). *Staphylococcus* was also found in the colostrum, although at a much lower abundance (1.52%), and was particularly depleted in BM compared with NS (Supplementary Figure S1C). Thus, the origin and types of *Staphylococcus* in BM and NS are worthy of further study.

The composition of the microbiota in reports varies widely, suggesting that the purpose of the studies, geographical location (Kumar et al., 2016; Li et al., 2017; Ding et al., 2019), ethnic differences, sampling volunteers (Ruiz et al., 2019), collection methods, and methodological differences (Sakwinska et al., 2016) may influence the results.

The microbiota of BM has been hypothesized to originate from commensal and potential probiotic bacteria colonized in the intestine of newborns. Through temperature gradient gel electrophoresis (TGGE) (Perez et al., 2007), pyrosequencing (Jost et al., 2012, 2014), and culture methods (Solis et al., 2010; Jost et al., 2012), *Bifidobacterium* strains, which are gut-associated obligate anaerobes, were found to be shared between maternal feces, BM, and neonatal feces, especially during the first week. Accordingly, our results also found that the relative abundance of *Bifidobacterium* at the genus level in colostrum was high (Figures 2C, 4B and Supplementary Figure S1D). Thus, our results support that mothers separated from their newborns should maintain lactation and feed colostrum to their babies in the NICU in order to promote the establishment of normal intestinal microbiota.

### Evidence of entero-mammary pathway of breast milk microbiota

Currently, it is still unclear how microbes reach the BM. Most previous studies have suggested the traditional contamination hypothesis (such as the mother’s skin and infant’s oral cavity). One suggested that mammary ducts become colonized by the infant oral microbiota during suckling, as the retrograde flow of BM into mammary ducts has been documented (Ramsay et al., 2004). However, other studies arrived at different conclusions. For example, buccal administration of colostrum to low-birth-weight newborns in the NICU changed their oral microbiota when compared to infants who were given standard care, suggesting that the BM might be responsible for colonizing the infant’s mouth (Sohn et al., 2016). Additionally, precolostrum already contains bacteria before suckling has occurred (Ruiz et al., 2019). Other studies have also suggested that bacteria from the skin (such as *Corynebacterium, Cutibacterium*, and *Staphylococcus*) colonize the mammary ducts. However, studies in recent years have suggested the revolutionary active migration hypothesis, which supports the existence of an entero-mammary pathway.

One of the main purposes of this study was to provide a practical and reliable basis for determining the source of bacteria in the BM. In order to avoid contamination by the infant’s oral cavity, we enrolled healthy, lactating women who were immediately separated from their newborn(s) after delivery in this study. The newborns did not suck the mother’s breast. To avoid the influence of skin microbiota, we washed the surrounding NS with sterile water, collected cleaning water, and then collected the BM after squeezing out the first drop of colostrum. Both the NS and BM samples were analyzed by 16S rRNA gene sequencing targeting the V3–V4 region to compare the composition differences between the microbiota.

Our results showed that there were shared microorganisms between the BM and NS microbiota. As shown in the Venn diagram, there were 170 overlapping OTUs (Supplementary Figure S1A) and no significant OTUs in the Volcanic map (Supplementary Figure S1B), as well as no significant bacterial classification level in the Manhattan plot (Supplementary Figure S1C). However, there were also significant differences in the microbiota diversity between BM and NS. The Venn diagram displayed 111 OTUs unique to BM and 87 unique to NS, while 77 OUTs were depleted and 107 were enriched in BM compared with NS through a Volcanic map (Supplementary Figure S1B) for advanced differential abundance analysis. PCoA for the β diversity demonstrated two different populations, BM and NS, in the principal coordinate PCo1, explaining 21.49% of the variance (Figure 3A). The unique microbiota in BM was screened by LEfSe analysis. Anaerobic bacteria found in the colostrum (BM), which are not found on the skin (Aakko et al., 2017; Damaceno et al., 2017; Williams et al., 2017; Parnanen et al., 2018), include *Bifidobacterium* at the genus level, *Enterobacteriaceae* at the family level, *Lactobacillales* and *Pseudomonadales* at the order level, *Actinobacteria* and *Gammaproteobacteria* at the class level, and *Proteobacteria* at the phylum level. Similar to our results regarding *Lactobacillales* at the order level in the BM, a study displayed that Lactobacillus present in BM are genotypically different from those detected on the skin within individuals (Martin et al., 2007b). While in NS, the predominance of *Staphylococcus* at the genus level was universally observed in other studies (Solis et al., 2010; Jost et al., 2013).

Excluding neonatal oral contamination, breast skin contamination, and microbiota shared by the NS, the source of bacteria in the breast is quite amazing. As many of the bacteria found in BM can also be found in the intestine, it is plausible that an entero-mammary pathway exists, that is, intestinal organisms, or their DNA, can be transferred from the intestine to the mammary ducts (Zimmermann and Curtis, 2020). Gut-associated obligate anaerobic genera, like *Bifidobacterium* and *Lactobacillales*, have been identified by pyrosequencing to be shared among maternal feces, BM, and neonatal feces (Jost et al., 2014; Cortes-Macias et al., 2021).

Therefore, the entero-mammary pathway hypothesis of BM microbiota might be able to explain this. Our results of unique taxa enriched in the BM, including *Bifidobacterium, Enterobacteriaceae, Lactobacillales*, and *Pseudomonadales*, as well as *Gammaproteobacteria* and even *Proteobacteria*, were likely transferred from the intestine to the breast through the intestinal tract.

Previous studies have verified that when *Lactobacillus* is administered as a probiotic to women, the same strain can be identified in BM (Jimenez et al., 2008; Abrahamsson et al., 2009). Translocation of bacteria from the intestine to the mammary glands is thought to mainly occur through gut-associated lymphoid tissues (Vazquez-Torres et al., 1999; Qutaishat et al., 2003) and involves dendritic cells and macrophages (Vazquez-Torres et al., 1999; Rescigno et al., 2001; Qutaishat et al., 2003). Moreover, the translocation of the microbiota has been reported to increase in pregnant or lactating women (Perez et al., 2007). A previous study reported the transmission of *Salmonella enterica* to infants through the mother’s BM. It was proposed that there is a biologically feasible mechanism of transport of *Salmonella* from the gastrointestinal tract to BM. *Salmonella* is resistant to the acidic environment of the stomach and usually invades or is phagocytosed by cells lining the Peyer’s patches in the small intestine. Specialized epithelial M cells overlying the lymphoid follicles of Peyer’s patches provide a portal of entry for *Salmonella enterica* Typhimurium. The pathogenicity island 1 of *S. enterica* Typhimurium was required to penetrate these intestinal epithelial M cells. *S. enterica* Typhimurium is transported from the gastrointestinal tract to the bloodstream by CD18-expressing phagocytic leukocytes and may use macrophages and dendritic cells as a conduit to deeper tissue (Qutaishat et al., 2003).

For non-pathogenic microorganisms, a previous study reported that dendritic cells may express tight junction proteins, which open the tight junctions between epithelial cells, allowing them to penetrate the gut epithelial monolayers to sample bacteria (Rescigno et al., 2001). In future studies, we will conduct a series of animal experiments to follow non-pathogenic bacteria in maternal rats to explore their effects on the BM microbiota, intestinal-breast pathway, establishment of intestinal microbiota, and intestinal immunity.

### Various factors had no effect on the microbiota diversity of colostrum (breast milk) but on differences in the unique bacteria in each group

In this study, various factors, including the newborn gender, delivery mode, parity, gestational age, and intrapartum antibiotics exposure (Supplementary Table S1), were considered to influence the microbiota diversity of colostrum (BM). Concerning the impact of the newborn gender, a study reported a lower diversity and richness in the microbiota of 3–4 months postpartum BM (mature milk) among mothers of male infants. We found no differences in the α and β indices of colostrum microbiota (Figures 4A,B). This may be due to the fact that BM has different lactation stages. The mothers of female newborns had a higher relative abundance of *Streptococcaceae* at the family level, which was the opposite result of another study which reported that mothers of male infants had a higher relative abundance of *Streptococcus* in mature milk (Williams et al., 2017). Mothers of male newborns in our study had a higher relative abundance of *Roseburia* at the genus level than those of female newborns (Supplementary Figure S2A). Gamez-Valdez et al. (2021) found that the BM microbiota was influenced by the infant gender in the obesity mother subgroup, with phylogenetic diversity of the female newborns more diverse than in males. Thus, the complex relationship between women’s obesity and gestational diabetes mellitus and the influence of newborn gender on the microbiota composition may be worthy of deep study.

We concluded that the delivery mode did not affect the α and β indices of the BM microbiota diversity (Figures 4C,D), which was similar to another study (Li et al., 2017). Furthermore, we found that women who delivered by CS had a higher relative abundance of *Bifidobacterium* and *E. coli-Shigella* in colostrum (BM) (Figure 4B). Thus, it could be suggested that CS might lead to increased intestinal permeability, enhanced bacterial translocation in the maternal gut, and consequently, a higher transfer of bacteria to the BM.

The conclusion of the studies showed that the effect of parity on the microbiota of BM included two aspects. One aspect was that the diversity in the BM microbiota was lower in infants who were first born as compared with those who had one or more siblings (Moossavi et al., 2019). The second aspect was that parity had no effect on the diversity of the BM microbiota. Our results were in favor of the latter (Figures 4E,F), and all microbiota were shared by parturient and primipara mothers.

It has been reported that women who receive IAP had a higher α diversity and richness in their BM microbiota, *Bifidobacterium* was not found (Hermansson et al., 2019), and a lower relative abundance of *Lactobacilli* was found (Soto et al., 2014). We found that IAP (cefazolin sodium was given intravenously to 14 mothers and cefuroxime sodium to 8 mothers) had no effect on the microbiota diversity of colostrum (BM) (Figures 4G,H). Though there was no *Bifidobacterium* in non-antibiotics BM as assessed by LDA scores, the BM was rich in *Lactobacillus* at the genus level (Supplementary Figure S2C). According to the American College of Obstetricians and Gynecologists (ACOG), IAP is recommended for all women undergoing C-section, and the first generation of cephalosporin, such as cefazolin sodium, is preferred. Fourteen mothers undergoing C-section in this study were given cephalosporin, including cefazolin sodium and cefuroxime sodium. The other eight mothers received cephalosporin prophylactically because of maternal *Streptococcus* B infection or premature rupture of membranes. They accepted antibiotics according to the guidelines. The half-life of cephalosporins is short, so the effect of these antibiotics on the BM microbiota was minimal by the time we collected the mothers’ BM on the third day after delivery (the average sampling days were 3 after delivery). Moreover, the type or level of antibiotics did not significantly affect the diversity of the colostrum microbiota. Additionally, *Streptococcus* was not found in the colostrum of mothers infected with *Streptococcus* B, indicating that this pathogenic bacterium did not enter the BM, or at least, was not found in the colostrum after the administration of antibiotics.

A few studies have explored the influence of gestational age on the BM microbiota (Khodayar-Pardo et al., 2014; Urbaniak et al., 2016). One study investigated a higher relative and absolute abundance of *Bifidobacterium* in women who delivered at full-term (Khodayar-Pardo et al., 2014), which was the same as our results in colostrum (BM) (Supplementary Figure S2D). Otherwise, we found no effect of gestational age on the microbiota composition of colostrum (BM) (Figures 4I,J), which was consistent with another study (Urbaniak et al., 2016).

As the first attempt to explore the microbiota of BM, we only analyzed the colostrum and NS microbiota in this study, without the collection and analysis of stool from mothers. Therefore, we will expand the sample number and continuously explore the microbiota changes in colostrum, transitional milk, and mature BM compared to the maternal stool to provide further evidence regarding the origin of the BM microbiota. Overall, illuminating the potential factors affecting the differential bacterial taxa of the BM microbiota, such as delivery mode, gestational age, and IAP, was meaningful and allows for in-depth research in the future.

## Conclusion

Our study further substantiates the presence and diversity of a microbiota specific to colostrum (BM) among mothers separated from their newborns. Furthermore, the comparison of the microbiota diversity between BM and NS provides reliable evidence for verifying the entero-mammary bacterial translocation hypothesis. According to our results of probiotics, such as *Bifidobacterium* in colostrum, and no effect of intrapartum antibiotics on microbiota abundance, we support mothers to maintain lactation and pump BM for newborns in NICU.

However, our results need to be further studied for confirmation. The impact of the BM microbiota on neonatal gut microbiota establishment, immunity function, and health consequences are worthy of further study.

## Data availability statement

The datasets presented in this study can be found in online repositories. The names of the repository/repositories and accession number(s) can be found below: NCBI—PRJNA848210.

## Ethics statement

The studies involving human participants were reviewed and approved by the Shenzhen Luohu Maternity and Child Healthcare Hospital (No. LL2022051337). The patients/participants provided their written informed consent to participate in this study. Written informed consent was obtained from the individual(s) for the publication of any potentially identifiable images or data included in this article.

## Author contributions

YD, ZHH, and RX performed the conception and design of the work and drafted the article. YD, QQ, and LW collected the samples and the data. ZOH and XW performed the data analysis and interpretation. ZOH and GJ performed the critical revision of the article. XW and GJ performed the final approval of the version to be published. All authors contributed to the article and approved the submitted version.

## Supplementary material

Supplementary Figure S1 Supplementary methods to describe the differences in the microbiota between BM (colostrum) and **(A)** A Venn diagram representing the overlapping and unique OTUs between BM and NS. **(B)** Taxonomic and functional characteristics of differential bacteria between the BM and NS microbiota. **(C)** Manhattan plot showing OTUs enriched in the BM as compared to NS. Each triangle represents a sing out OTU. OTUs enriched in BM or NS are represented by filled or empty triangles, respectively (FDR < 0.005, Wilcoxon rank-sum test). OTUs are arranged in taxonomic order and colored according to the genus. The size of the triangle represents the abundance of OTUs. **(D)** Linear discriminant analysis (LDA) scores computed for differentially abundant taxa in the microbiomes of NS (blue) and BM (red). Length indicates effect size associated with a taxon; *P* = 0.05 for the Kruskal–Wallis *H*-test; LDA score = 4.

Supplementary Figure S2 Differential bacterial taxa in the microbiome of NS (green) and BM (red) in different groups by linear discriminant analysis (LDA) scores. The length indicates the effect size associated with a taxon; *p* = 0.05 for the Kruskal–Wallis *H*-test; LDA score = 3.

Supplementary Table S1 The demographics of the participants.
