# Supplementary figures and images for: Comparative study on the microbiota of colostrum and nipple skin from lactating mothers separated from their newborn at birth in China
Source: Front Microbiol. 2022 Oct 3;13:932495. doi: 10.3389/fmicb.2022.932495 (PMC9574262; doi:10.3389/fmicb.2022.932495)

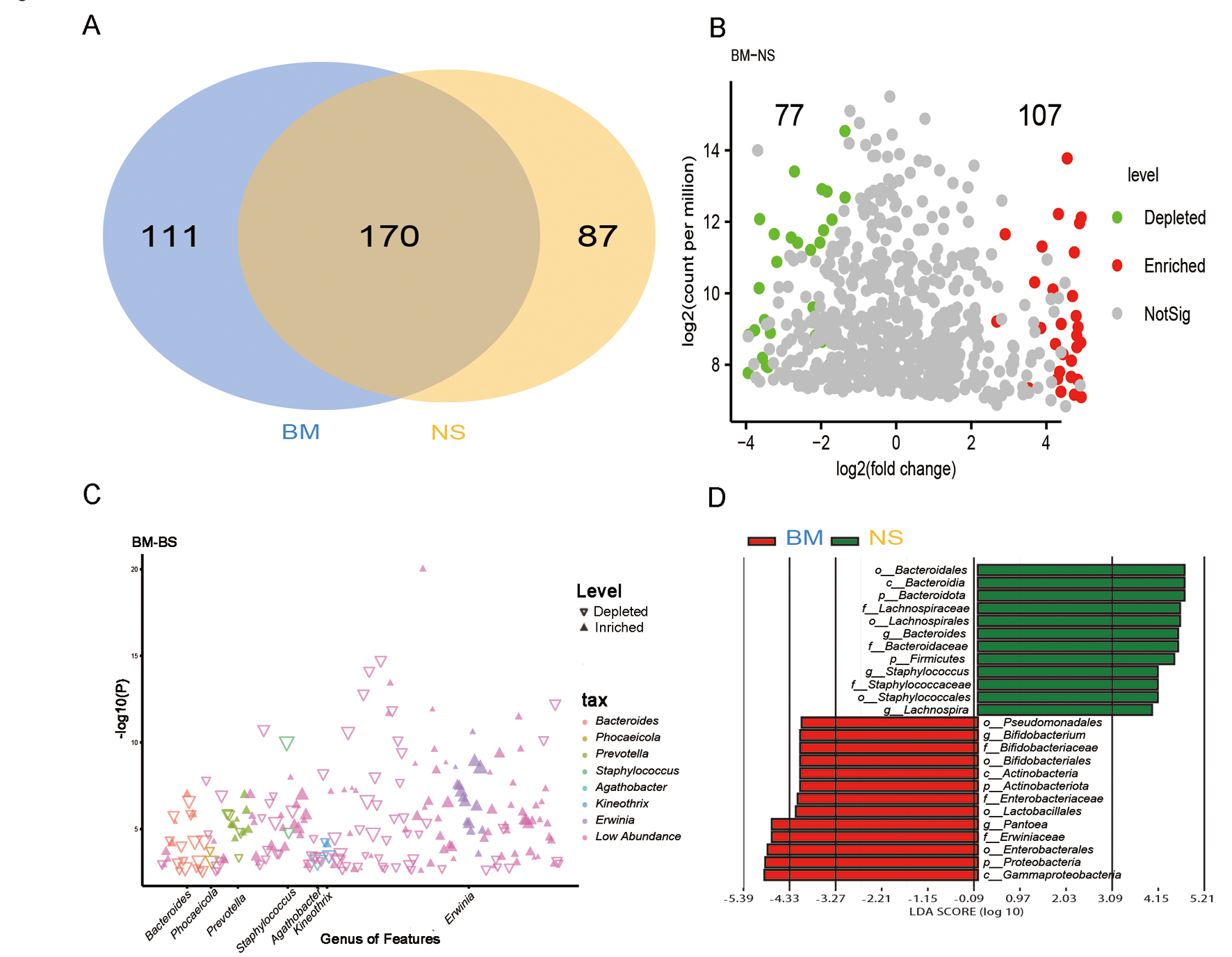

Supplement: Supplementary Figure S1 — Supplementary methods to describe the differences in the microbiota between BM (colostrum) and (A) A Venn diagram representing the overlapping and unique OTUs between BM and NS. (B) Taxonomic and functional characteristics of differential bacteria between the BM and NS microbiota. (C) Manhattan plot showing OTUs enriched in the BM as compared to NS. Each triangle represents a sing out OTU. OTUs enriched in BM or NS are represented by filled or empty triangles, respectively (FDR < 0.005, Wilcoxon rank-sum test). OTUs are arranged in taxonomic order and colored according to the genus. The size of the triangle represents the abundance of OTUs. (D) Linear discriminant analysis (LDA) scores computed for differentially abundant taxa in the microbiomes of NS (blue) and BM (red). Length indicates effect size associated with a taxon; P = 0.05 for the Kruskal–Wallis H-test; LDA score = 4. [file Image_1.TIF]

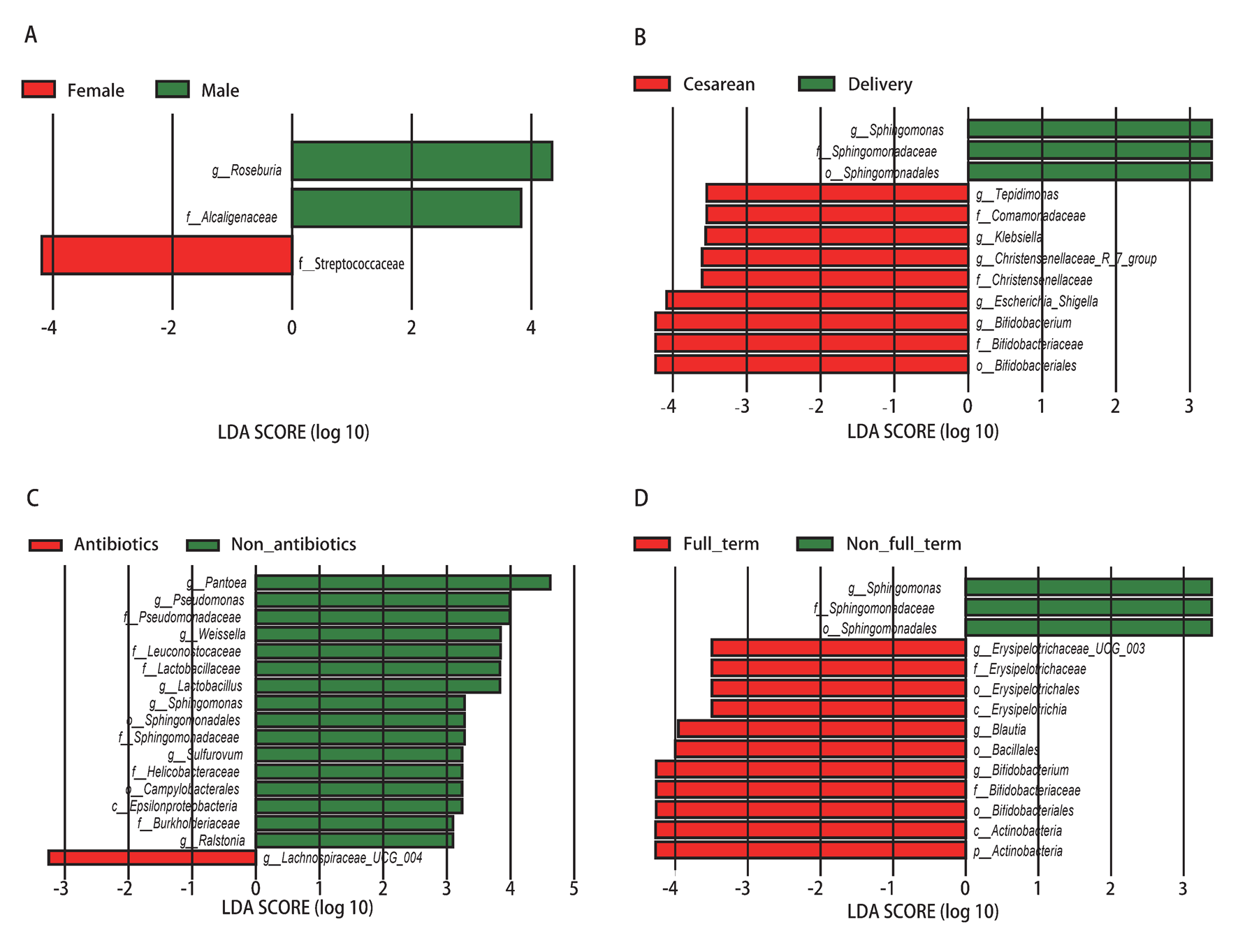

Supplement: Supplementary Figure S2 — Differential bacterial taxa in the microbiome of NS (green) and BM (red) in different groups by linear discriminant analysis (LDA) scores. The length indicates the effect size associated with a taxon; p = 0.05 for the Kruskal–Wallis H-test; LDA score = 3. [file Image_2.TIFF]
