# Supplementary material for: Comparative study on the microbiota of colostrum and nipple skin from lactating mothers separated from their newborn at birth in China
Source: Front Microbiol. 2022 Oct 3;13:932495. doi: 10.3389/fmicb.2022.932495 (PMC9574262; doi:10.3389/fmicb.2022.932495)
Supplement: Supplementary Table S1 — The demographics of the participants. [file Table_1.DOCX]

Supplementary Material Table 1. The demographics of the participants.

| **Mother number** | **Age**  **(years)** | **Parity** | **Gestational**  **Age (weeks)** | **Delivery**  **mode** | **Use of intrapartum antibiotics during delivery** | **Infant gender** | **Reasons for mother-infant separation** | **Sampling days after birth (days)** |
| --- | --- | --- | --- | --- | --- | --- | --- | --- |
| 1 | 42 | 3 | 39 | Vaginal | No | M | pulmonary sequestration on the left side of the newborn | 3 |
| 2 | 33 | 1 | 39 | C-section | Yes  (Cefazolin Sodium) | F | [Premature](javascript:;) [rupture](javascript:;) [of](javascript:;) [membranes](javascript:;) | 4 |
| 3 | 30 | 1 | 39 | Vaginal | No | M | II° amniotic fluid pollution | 3 |
| 4 | 28 | 1 | 40 | Vaginal | Yes  (Cefazolin Sodium) | F | [Premature](javascript:;) [rupture](javascript:;) [of](javascript:;) [membranes](javascript:;) | 2 |
| 5 | 25 | 1 | 39 | Vaginal | Yes  (Cefazolin Sodium) | M | [Premature](javascript:;) [rupture](javascript:;) [of](javascript:;) [membranes](javascript:;) | 1 |
| 6 | 29 | 2 | 39 | Vaginal | No | M | Maternal *Streptococcus* B infection | 1 |
| 7 | 29 | 3 | 38 | C-section | Yes  (Cefazolin Sodium) | M | Aspiration of amniotic fluid | 2 |
| 8 | 29 | 1 | 35 | Vaginal | No | F | [Premature](javascript:;) [infant](javascript:;) | 2 |
| 9 | 29 | 1 | 37 | C-section | Yes  (Cefazolin Sodium) | M | Low weight of twins | 3 |
| 10 | 30 | 3 | 35 | C-section | Yes  (Cefazolin Sodium) | M | [Premature](javascript:;) [infant](javascript:;) | 2 |
| 11 | 29 | 1 | 36 | Vaginal | Yes  (Cefazolin Sodium) | F | [Premature](javascript:;) [infant](javascript:;) | 3 |
| 12 | 26 | 2 | 35 | Vaginal | No | M | [Premature](javascript:;) [infant](javascript:;) | 2 |
| 13 | 28 | 2 | 38 | Vaginal | No | F | Maternal *Streptococcus* B infection | 2 |
| 14 | 30 | 2 | 39 | Vaginal | Yes  (Cefuroxime Sodium) | M | [Premature](javascript:;) [rupture](javascript:;) [of](javascript:;) [membranes](javascript:;) | 2 |
| 15 | 29 | 1 | 35 | C-section | Yes  (Cefazolin Sodium) | F | [Premature](javascript:;) [delivery](javascript:;) | 3 |
| 16 | 32 | 1 | 41 | C-section | Yes  (Cefazolin Sodium) | F | Amniotic fluid pollution | 4 |
| 17 | 32 | 1 | 39 | C-section | Yes  (Cefuroxime Sodium) | M | [Placental](javascript:;) [abruption](javascript:;) | 4 |
| 18 | 22 | 1 | 34 | Vaginal | No | M | [Premature](javascript:;) [delivery](javascript:;) | 3 |
| 19 | 24 | 1 | 40 | Vaginal | Yes  (Cefuroxime Sodium) | M | [Partus](javascript:;) [serotinus](javascript:;) | 5 |
| 20 | 25 | 1 | 30 | Vaginal | Yes  (Cefuroxime Sodium) | F | [Premature](javascript:;) [delivery](javascript:;) | 1 |
| 21 | 38 | 1 | 37 | Vaginal | No | M | [Premature](javascript:;) [rupture](javascript:;) [of](javascript:;) [membranes](javascript:;) | 4 |
| 22 | 27 | 1 | 38 | C-section | Yes  (Cefazolin Sodium) | M | [Premature](javascript:;) [rupture](javascript:;) [of](javascript:;) [membranes](javascript:;) | 4 |
| 23 | 25 | 1 | 40 | Vaginal | No | F | Amniotic fluid turbidity | 5 |
| 24 | 25 | 1 | 39 | C-section | Yes  (Cefazolin Sodium) | F | Maternal *Streptococcus* B infection | 4 |
| 25 | 38 | 2 | 39 | C-section | Yes  (Cefazolin Sodium) | M | Mature low birth weight infants | 4 |
| 26 | 22 | 1 | 38 | Vaginal | Yes  (Cefuroxime Sodium) | M | Amniotic fluid pollution | 3 |
| 27 | 35 | 2 | 38 | C-section | Yes  (Cefazolin Sodium) | F | Maternal *Streptococcus* B infection | 2 |
| 28 | 29 | 1 | 37 | Vaginal | Yes  (Cefuroxime Sodium) | M | Amniotic fluid pollution | 3 |
| 29 | 33 | 2 | 40 | C-section | Yes  (Cefazolin Sodium) | M | [Prolapse](javascript:;) [of](javascript:;) [cord](javascript:;) | 3 |
| 30 | 27 | 1 | 40 | C-section | Yes  (Cefuroxime Sodium) | F | Amniotic fluid pollution | 4 |
| 31 | 30 | 1 | 39 | Vaginal | Yes  (Cefuroxime Sodium) | M | Acute chorionic inflammation | 3 |
